# Conversational Analyses of Older Adults’ Social Engagement with AI-Driven Socially Assistive Robots

**DOI:** 10.1093/geroni/igaf122.1743

**Published:** 2025-12-31

**Authors:** Othelia Lee, Narae Choi, Do-Hyung Park

**Affiliations:** The University of North Carolina at Charlotte, Charlotte, North Carolina, United States; Kookmin University, Seoul, Republic of Korea; Kookmin University, Seoul, Republic of Korea

## Abstract

**Objectives:**

The digital divide and limited digital literacy hinder technology adoption among older adults in low-resource communities. This study examines how socially assistive robots (SARs) can facilitate social engagement and emotional well-being among older adults by analyzing conversational interactions with the AI-driven Hyodol SAR.

**Methods:**

Multimodal data—including log-based usage patterns and conversational voice data—were collected through SAR-embedded sensors. Pre- and post-surveys gathered information on demographics and health status. Human-robot conversations were categorized into nine emotional and topical categories and six types of activity participation. To explore user engagement patterns, K-means clustering was applied to identify distinct user personas.

**Results:**

Among participants, 44.6% engaged in conversations with the SARs, with 30.2% discussing their activity participation. Three personas emerged: Social Butterflies (n = 19, 28.35%) maintained balanced engagement in social and personal activities, with positive emotional exchanges but limited long-term impact on well-being. Lone Wolves (n = 28, 41.79%) had low social engagement, yet showed notable improvements in emotional well-being through conversational interactions with the SAR. Emotional Peacocks (n = 20, 29.85%) displayed high emotional and sensory engagement with the SAR, demonstrating the greatest reduction in loneliness among the three groups.

**Discussion:**

Findings suggest that SARs helped mitigate social isolation, especially among older adults with limited social engagement. Furthermore, the integration of large language models with SAR technology enables autonomous, dialogue-based AI companions delivering personalized, emotion-sensitive interactions. Future research should explore adaptive AI learning models, ethical considerations in caregiving, and the long-term effects of SAR-facilitated engagement on well-being.

